# Sex‐related differences in amyotrophic lateral sclerosis: A 2‐[ ^18^F]FDG‐PET study

**DOI:** 10.1111/ene.16588

**Published:** 2024-12-10

**Authors:** Antonio Canosa, Alessio Martino, Umberto Manera, Alessandro Giuliani, Rosario Vasta, Francesca Palumbo, Maurizio Grassano, Silvia Daniela Morbelli, Matteo Pardini, Agostino Chiaravalloti, Orazio Schillaci, Klaus Leonard Leenders, Rosalie Vered Kogan, Giulia Polverari, Grazia Zocco, Francesca Di Pede, Filippo De Mattei, Sara Cabras, Enrico Matteoni, Cristina Moglia, Andrea Calvo, Adriano Chiò, Marco Pagani

**Affiliations:** ^1^ ALS Centre, ‘Rita Levi Montalcini’ Department of Neuroscience University of Turin Turin Italy; ^2^ Azienda Ospedaliero‐Universitaria Città della Salute e della Scienza di Torino Neurology Unit 1U Turin Italy; ^3^ Institute of Cognitive Sciences and Technologies, C.N.R Rome Italy; ^4^ Department of Business and Management LUISS University Rome Italy; ^5^ Environment and Health Department Istituto Superiore di Sanità Rome Italy; ^6^ Department of Medical Sciences University of Turin Turin Italy; ^7^ Azienda Ospedaliero‐Universitaria Città della Salute e della Scienza di Torino Nuclear Medicine Unit Turin Italy; ^8^ Department of Neuroscience, Rehabilitation, Ophthalmology, Genetics, Maternal and Child Health (DINOGMI) University of Genoa Genoa Italy; ^9^ IRCCS Ospedale Policlinico San Martino Genoa Italy; ^10^ Department of Biomedicine and Prevention University of Rome ‘Tor Vergata’ Rome Italy; ^11^ IRCCS Neuromed Pozzilli Italy; ^12^ Department of Neurology University of Groningen, University Medical Center Groningen Groningen The Netherlands; ^13^ Department of Nuclear Medicine and Molecular Imaging University of Groningen, University Medical Center Groningen Groningen The Netherlands; ^14^ Positron Emission Tomography Centre AFFIDEA‐IRMET S.p.A Turin Italy; ^15^ Center for Neuroscience University of Camerino Camerino Italy; ^16^ Neuroscience Institute of Turin (NIT) Turin Italy; ^17^ Department of Medical Radiation Physics and Nuclear Medicine Karolinska University Hospital Stockholm Sweden

**Keywords:** 2‐[^18^F]FDG‐PET, amyotrophic lateral sclerosis, brain connectivity, brain metabolism, sex differences

## Abstract

**Purpose:**

We investigated sex‐related brain metabolic differences in Amyotrophic Lateral Sclerosis (ALS) and healthy controls (HC).

**Methods:**

We collected two equal‐sized groups of male (m‐ALS) and female ALS (f‐ALS) patients (*n* = 130 each), who underwent 2‐[^18^F]FDG‐PET at diagnosis, matched for site of onset, cognitive status and King's stage. We included 168 age‐matched healthy controls, half female (f‐HC) and half male (m‐HC). We compared brain metabolism of males and females separately for ALS and HC, including age as covariate. A differential network analysis was performed to evaluate brain connectivity.

**Results:**

M‐ALS showed relative hypometabolism of bilateral medial frontal, parietal and occipital cortices, and left temporal cortex, compared to f‐ALS. In node‐wise comparison, f‐ALS showed significantly higher connectivity in right middle cingulate cortex and left superior and medial frontal gyrus. In HC we did not find any sex‐related differences.

**Conclusion:**

Sex resulted a major determinant of brain metabolism and connectivity in ALS patients.

## INTRODUCTION

Amyotrophic Lateral Sclerosis (ALS) is a fatal neurodegenerative disease affecting both sexes, probably determined by a complex interaction of genes and environmental factors [1]. Both motor and cognitive features that affect disease progression and overall survival are influenced by age, sex and genetic background [2] across different populations [3, 4]. Epidemiological data from population‐based registers point out that ALS is slightly more frequent in males compared to females but the ratio seems to decrease over time, possibly due to exogenous factors with a differential effect on the two sexes. As regards the site of onset, bulbar onset is more frequent in females [5]. Moreover, women have a later median age at onset compared with men [6].The underlying bases of sex differences in ALS have been studied, both in animal models and in human neuropathological cases. In a rat model, male sex was reported to be associated with an earlier onset of motor impairment compared to females, but the disease progression was similar in the two sexes [7]. Further studies investigating the effect of ovariectomy and sexual hormones in rat and mouse models showed conflicting results [8, 9, 10].

As regards data from human neuropathological studies, they did not report any sex‐related difference [11]. In vivo neuroimaging studies on this issue are limited, but Magnetic Resonance Imaging (MRI) data show differences between male and female ALS patients in terms of cortical atrophy and structural and functional connectivity [12, 13].

Conversely, evidence coming from studies on normal ageing and other neurological diseases like Alzheimer's disease (AD) pointed out that sex is one of the major determinants of functional and structural changes occurring over time due to physiological ageing or neurodegeneration [14, 15, 16].

The comprehension of sex‐related differences in ALS should be oriented to face the issue of disappointing results of clinical trials in the context of a precision medicine approach. Indeed, disease heterogeneity is one of the major pitfalls hampering the search for novel therapies [17].

In the present study, using a large 2‐[^18^F]fluoro‐2‐deoxy‐D‐glucose‐Positron‐Emission Tomography (2‐[^18^F]FDG‐PET) dataset of ALS patients and healthy subjects, we focused on the disease‐related metabolic brain differences associated with sex, adjusting for other factors contributing to phenotypic variability.

## METHODS

### Participants

We screened for eligibility all consecutive patients diagnosed with definite, probable and probable laboratory‐supported ALS according to El Escorial revised diagnostic criteria [18] between 2009 and 2019 at the ALS Expert Centre of Turin, Italy, who underwent brain 2‐[^18^F]FDG‐PET at the time of diagnosis. The following demographic and clinical characteristics were collected: age at PET, sex (female/male), site of onset (spinal/bulbar), ALS Functional Rating Scale‐Revised (ALSFRS‐R), ΔALSFRS‐R, King's stage at PET, cognitive status and presence of mutations in *SOD1*, *TARDBP*, *FUS* and *C9ORF72* genes. ΔALSFRS‐R was calculated as the number of points lost per month from disease onset to PET scan. King's staging was calculated from the ALSFRS‐R score according to a published algorithm [19], and King's stages 4a and 4b were combined as stage 4. As for cognitive assessment, we used a test battery reported elsewhere [20] and revised diagnostic criteria for frontotemporal spectrum disorders in ALS [21]. The methodology of genetic analysis has been reported in a previous study [22].

Out of 657 potentially eligible subjects, we excluded patients carrying mutations in the above mentioned genes. Subsequently, we randomly collected two equal‐sized groups of male and female patients, respectively, matched for site of onset, cognitive status (considered as binary, i.e. normal/impaired) and King's stage, in order to achieve two homogenous samples according to motor and cognitive features.

For a more comprehensive evaluation of eventual brain metabolic and connectivity differences between males and females associated with ALS, we included a healthy control sample (HC) collected from four centres: ALS Expert Centre of Turin, Italy; Nuclear Medicine Unit, Department of Health Sciences, University of Genoa, Italy; Department of Biomedicine and Prevention, University of Rome Tor Vergata, Italy; Department of Nuclear Medicine and Molecular Imaging, University Medical Center Groningen, University of Groningen, The Netherlands. Male and female HC were matched for age (± 5 years) and centre of origin. They had no history of neurological disorders, and a normal neurological examination, with brain PET scan reported as normal by a nuclear medicine physician. Data of HC were anonymized according to the European regulations for the protection of privacy.

### 2‐[ ^18^F]FDG‐PET image acquisition and pre‐processing

Brain 2‐[^18^F]FDG‐PET was performed according to published guidelines [23]. Patients fasted at least 6 h before the exam. Blood glucose was <7.2 mmol/L in all cases before the procedure. After a 20 min rest, about 185 MBq of 2‐[^18^F]FDG was injected. The acquisition started 60 min after the injection. PET/CT scans were performed on a Discovery ST‐E System (General Electric). Brain CT and PET scan were sequentially acquired, the former being used for attenuation correction of PET data. The PET images were reconstructed with four iterations and 28 subsets with an initial voxel size of 2.34 × 2.34 × 2.00 mm and data were collected in 128 × 128 matrices.

Images were spatially normalized to a customized brain 2‐[^18^F]FDG‐PET template [24] and subsequently smoothed with a 10 mm filter in MATLAB R2018b (MathWorks). Intensity normalization was performed at individual level averaging each voxel for the mean value of the whole brain.

### Statistical analysis

Descriptive statistics were performed using the Mann–Whitney *U* test and the chi‐square test, as appropriate. For PET analyses we compared male and female ALS patients employing the two‐sample *t*‐test model of SPM12, including age at PET as covariate. We compared male and female HC using the same SPM12 model. Moreover, we compared male patients and male HC on one side and female patients and female HC on the other side, adjusting for age. In all these group comparisons the height threshold was set at *p* < 0.05 FWE‐corrected.

Finally, we assessed the relationship between brain metabolism and motor staging and cognitive status, respectively. The methods are described in the Data S1.

In all the analyses, only clusters containing ≥100 contiguous voxels were considered significant. Brodmann areas (BAs) were identified at a 0–2 mm range from the Talairach coordinates of the SPM output isocentres corrected by Talairach Client (http://www.talairach.org/index.html).

### Differential network analysis

In order to further characterize eventual metabolic differences between male and female subjects, a differential network analysis has been performed. Starting from each patient, 94 brain Volumes of Interest (VOIs) and MetaVOIs have been extracted [25], along with their respective normalized uptake values. Differential network analysis was applied between the two networks built starting from the set of male ALS patients and female ALS patients in order to quantify the differences in terms of their respective connectivity. Each network was constructed by considering only the patients belonging to either groups (i.e. only male ALS or only female ALS) and then by evaluating the pairwise Pearson correlation coefficient between their respective 94 (Meta)VOIs and finally retaining only connections between regions whether the absolute value of the Pearson correlation coefficient was ≥0.7 in order to account both positively‐ and negatively correlated regions. Once the networks have been built, differential network analysis was performed in order to assess to which extent the two groups differ in terms of connectivity [26]. In our case, we employed the degree of the nodes since such centrality measure quantifies the ‘hubness’ of a node according to the size of its neighbourhood (i.e. a node with more connections will have a larger degree value). To deepen our study, we performed two different tests: at network level (in order to quantify whether the average degree centrality differs between the two networks) and at node level (in order to quantify whether the degree centrality differs between each node of the two networks). In order to evaluate the soundness of the discrepancies in terms of connectivity (both at node level and network level) between the two groups, we evaluated their statistical significance against 1000 different configuration models. Briefly, a configuration model is a random network derived from the ‘original network’ in which the degree distribution is preserved, although nodes are randomly re‐wired. In this manner, we are able to evaluate whether the degree of each single node (and by extension, of the network) is due to chance or as output of the intrinsic complexity of ALS [27]. We have also repeated the differential network analysis as described above comparing female ALS versus female HC, male ALS versus male HC and, for the sake of completeness, between male HC and female HC.

## RESULTS

### Demographic and clinical characteristics of the study groups

We included in the analyses 130 male and 130 female ALS patients and 168 HC, 84 males and 84 females. The demographic and clinical characteristics of patients, and the demographic characteristics and the centre of origin of HC are reported in Table 1. The age at PET did not result significantly different between ALS males and females.

**TABLE 1:** Descriptive statistics of ALS patients and healthy controls, subdivided by sex.

	Female	Male	*p*
Healthy controls (*N* = 168)			
Age at 2‐[^18^F]FDG‐PET [years, median (IQR)]	62.0 (45.7–70.0)	63.0 (48.5–70.0)	0.537^a^
Centre of origin, *n* (%)			
ALS Expert Centre of Turin	11 (13.1)	11 (13.1)	1.000^b^
University of Genoa	14 (16.7)	14 (16.7)	
University of Rome Tor Vergata	41 (48.8)	41 (48.8)	
University Medical Center Groningen	18 (21.4)	18 (21.4)	
ALS patients (*N* = 260)			
Age at 2‐[^18^F]FDG‐PET [years, median (IQR)]	67.1 (57.2–73.1)	66.8 (58.1–73.0)	0.905^a^
ALSFRS‐R total score [median (IQR)]	39.5 (34.0–43.0)	40.5 (36.0–44.0)	0.275^a^
ΔALSFRS‐R [points lost per month, median (IQR)]	0.54 (0.35–0.91)	0.67 (0.34–1.08)	0.343^a^
Survival (years)	3.2 (2.7–3.6)	2.6 (2.4–2.9)	0.088^c^
Site of onset, *n* (%)			
Bulbar onset	43 (33.1)	43 (33.1)	1.000^b^
Spinal onset	87 (66.9)	87 (66.9)	
King's staging system, *n* (%)			
Stage 1	48 (36.9)	48 (36.9)	1.000^b^
Stage 2	42 (32.3)	42 (32.3)	
Stage 3	40 (30.8)	40 (30.8)	
Cognitive status, *n* (%)			
ALS‐CN	80 (61.5)	80 (61.5)	0.996^b^
ALS‐bi	17 (13.1)	16 (12.3)	
ALS‐ci	18 (13.8)	19 (14.6)	
ALS‐cbi	7 (5.4)	8 (6.2)	
ALS‐FTD	8 (6.2)	7 (5.4)	
Total, *n* (%)	130 (100.0)	130 (100.0)	

Abbreviations: ALS‐bi, ALS with behavioural impairment; ALS‐cbi, ALS with cognitive and behavioural impairment; ALS‐ci, ALS with cognitive impairment; ALS‐CN, ALS with normal cognitive function; ALSFRS‐R, Amyotrophic Lateral Sclerosis Functional Rating Scale–Revised; ALS‐FTD, ALS with Frontotemporal Dementia; IQR, interquartile range.

^a^ Mann–Whitney *U* test.

^b^ Chi‐square test.

^c^ Log‐rank test.

### 2‐[ ^18^F]FDG‐PET data

**FIGURE 1:**
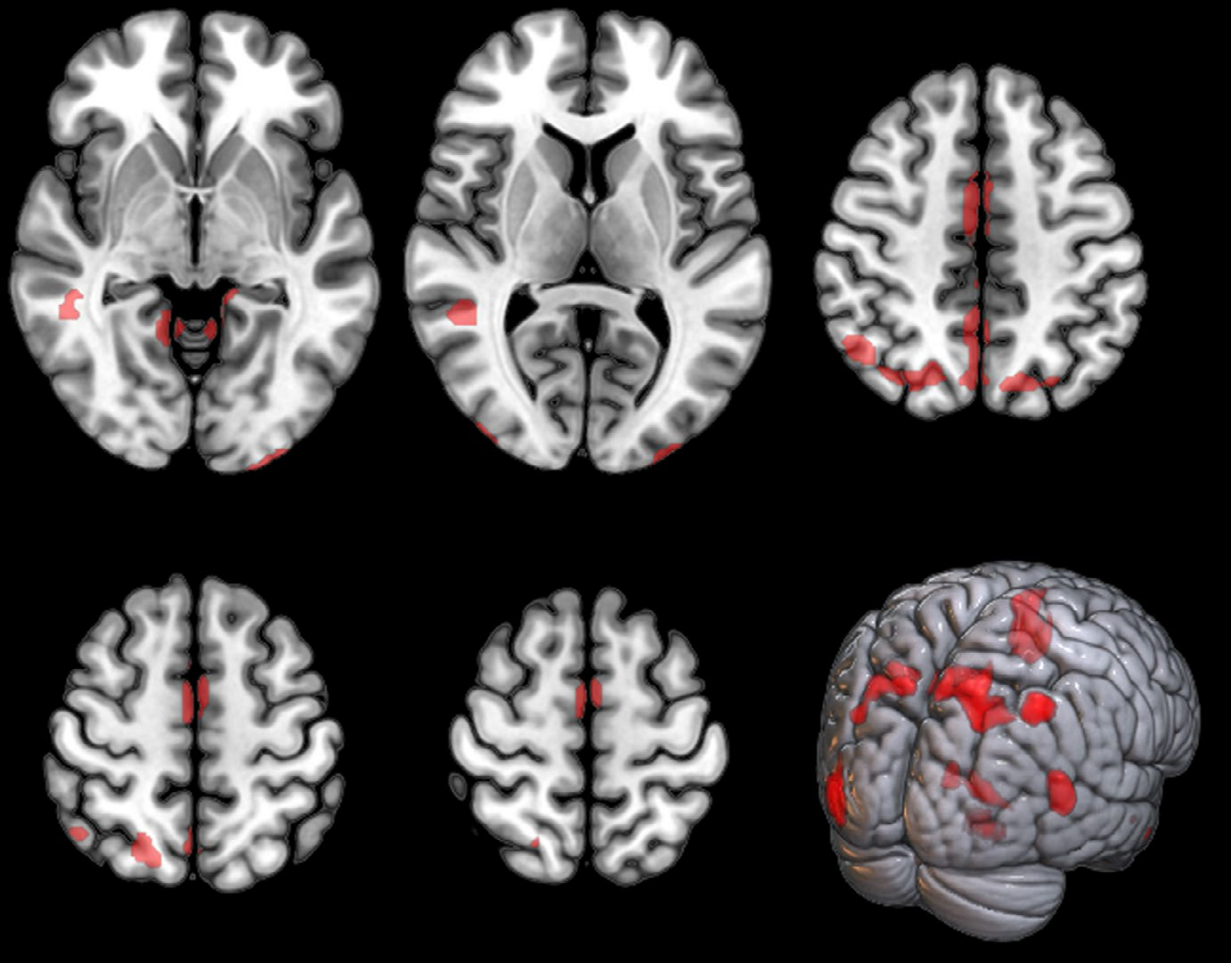
Clusters of relative hypometabolism of male as compared to female ALS patients are marked in red and are reported on axial sections of a brain magnetic resonance imaging template and on the brain surface of a glass brain rendering (bottom right).

As compared to females, male ALS patients showed clusters of relative hypometabolism including bilateral medial frontal, parietal and occipital cortices, and left temporal cortex (Figure 1, Table 2). In the comparison between male and female HC no significant difference emerged.

**TABLE 2:** Clusters of relative hypometabolism of male as compared to female ALS patients.

p(FWE‐corrected)	Cluster extent	Z‐score	Talairach coordinates (x, y, z)			Side	Brain Region	BA
0.000	400	4.95	−14	−35	−5	L	Parahippocampal Gyrus	30
0.000	228	4.58	−30	−94	21	L	Middle Occipital Gyrus	19
0.000	313	5.83	44	−89	10	R	Middle Occipital Gyrus	19
0.000	934	5.62	2	−48	45	R	Precuneus	7
		5.44	48	−54	49	R	Inferior Parietal Lobule	40
		4.96	26	−68	44	R	Superior Parietal Lobule	7
0.000	778	5.23	2	−2	39	R	Cingulate Gyrus	24
		5.12	0	−3	57	L	Medial Frontal Gyrus	6
0.000	195	5.06	−14	−68	48	L	Precuneus	7
		4.93	−28	−68	44	L	Precuneus	19
		4.80	−36	−72	29	L	Middle Temporal Gyrus	39

Abbreviations: BA, Brodmann Area; L, Left; R, Right.

In the comparison with male HC, male ALS patients showed a relative hypometabolism in left frontal cortex, left parahippocampal gyrus, left cerebellar culmen, left cuneus and right posterior cingulate cortex (Table S1), and a relative hypermetabolism in right precentral gyrus and right middle occipital gyrus (Table S2).

In the comparison with female HC, female ALS patients showed a relative hypometabolism in bilateral frontal cortex and left pallidum (Table S3), and a relative hypermetabolism in right postcentral gyrus, right middle occipital gyrus and right superior temporal gyrus (Table S4).

Evaluating the relationship between brain metabolism and cognitive status in ALS patients revealed similar results in males and females: the more severe the cognitive impairment, the lower the frontal metabolism and the higher the cerebellar metabolism (Figures S1–S4). Otherwise, for King's stage, we observed an increase in metabolism of the corticospinal tracts and the brainstem with increasing stage only in male ALS subjects (Figure S5).

### Differential network analysis

**FIGURE 2:**
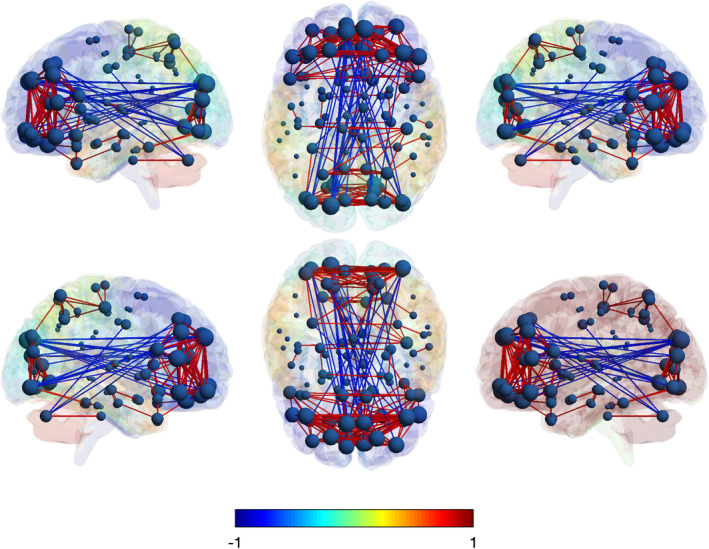
Whole‐brain metabolic connectivity of ALS females is represented on a 3D brain template. The spheres represent the nodes: The higher the size, the higher the metabolic connectivity. Red connections stand for positive correlations, the blue ones for negative correlations.

**FIGURE 3:**
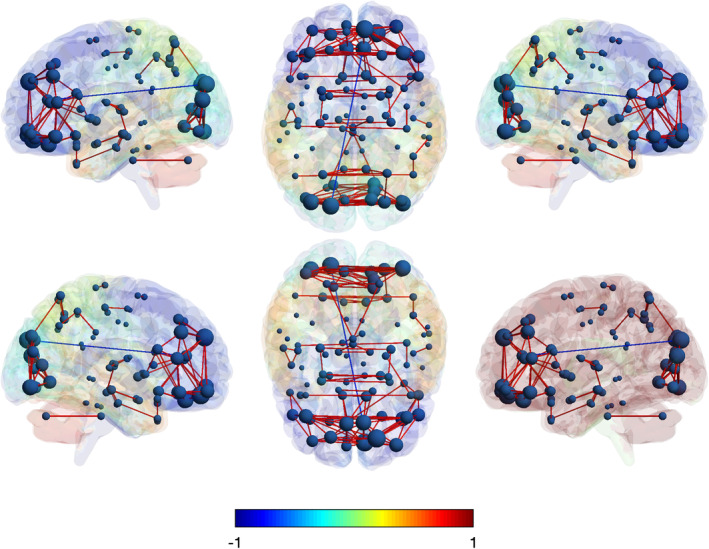
Whole‐brain metabolic connectivity of ALS males is represented on a 3D brain template. The spheres represent the nodes: The higher the size, the higher the metabolic connectivity. Red connections stand for positive correlations, the blue ones for negative correlations.

The degree centrality between networks pertaining to male and female ALS subjects emerged to be statistically significant (test statistics = 1.8448, adj. *p* = 0.004). In Table 3 we summarize the results of the node‐wise comparison between male and female ALS patients, where two regions showed significantly increased connectivity in females compared to males (i.e. right middle cingulate cortex and left superior and medial frontal gyrus; avg. test statistics = 8.2785, avg. adj. *p* = 0.047). Right middle cingulate gyrus showed increased connectivity with bilateral cerebellum (negative correlation) and contralateral middle cingulate gyrus (positive correlation). Left superior medial frontal gyrus displayed significantly increased connectivity with calcarine cortex and inferior, middle and superior occipital cortex (negative correlation), and with anterior cingulate cortex as well as orbital, middle and superior frontal cortex (positive correlation). In Figures 2 and 3 we report whole‐brain connectivity in female and male ALS patients, respectively.

**TABLE 3:** Results of the node‐wise comparison between male and female ALS patients.

Node	Test statistics	Nominal *p*‐value	*q*‐value	Female degree centrality score	Male degree centrality score
Right Middle Cingulate Gyrus	2.156	0.001	0.047	2.156	0
Left Superior Medial Frontal Gyrus	14.401	0.001	0.047	15.979	1.578

By comparing female ALS versus female HC, the degree centrality between networks still showed statistical differences (test statistics = 3.2611, adj. *p* = 0.001). At node level, we show in Table S5 the 30 (meta)VOIs in which there is a statistical significance (adj. *p* < 0.05) between female subjects.

In the comparison between male ALS and male HC, the degree centrality at network level showed statistical significance (test statistics = 1.0481, adj. *p* = 0.013) and the two (meta)VOIs in which we found a statistical significance in terms of their respective degree are reported in Table S6. In Figures S6 and S7 we report whole‐brain connectivity in female HC and male HC, respectively.

Finally, we did not find any differences between males and females in the control groups in terms of connectivity.

Table 4 summarizes the results of the comparison between ALS patients and healthy controls, stratified by sex, in terms of brain metabolism and brain connectivity.

**TABLE 4:** Brain metabolism and brain connectivity comparison between ALS patients and healthy controls, stratified by sex.

Side	Brain region	Gyrus/nucleus	Females		Males	
			Metabolism	Connectivity	Metabolism	Connectivity
L	Basal Ganglia	Pallidum	**↓**			
R	Basal ganglia	Pallidum				↑
L	Cerebellum	Cerebellar Culmen			**↓**	
L	Frontal	Inferior Frontal Gyrus	**↓**	**↑**	**↓**	
BIL	Frontal	Inferior Orbital Frontal Gyrus		**↑**		
BIL	Frontal	Medial Frontal Gyrus	**↓**			
L	Frontal	Middle Frontal Gyrus	**↓**	**↑**	**↓**	
R	Frontal	Middle Orbital Frontal Gyrus		**↑**		
R	Frontal	Precentral Gyrus		**↓**	**↑**	
L	Frontal	Rectus Gyrus		**↑**		
L	Frontal	Superior Frontal Gyrus	**↓**	**↑**	**↓**	
R	Frontal	Superior Frontal Gyrus	**↓**	**↑**		
BIL	Frontal	Superior Medial Frontal Gyrus		**↑**		
BIL	Frontal	Superior Orbital Frontal Gyrus		**↑**		
R	Limbic	Anterior Cingulum		**↑**		
R	Limbic	Middle Cingulum		**↑**		
L	Limbic	Posterior Cingulum				↓
BIL	Occipital	Calcarine Cortex		**↑**		
L	Occipital	Cuneus			**↓**	
L	Occipital	Inferior Occipital Gyrus		**↑**		
BIL	Occipital	Lingual Gyrus		**↑**		
L	Occipital	Middle Occipital Gyrus		**↑**		
R	Occipital	Posterior Cingulate			**↓**	
L	Occipital	Superior Occipital Gyrus		**↑**		
R	Occipital	Superior Occipital Gyrus		**↓**		
R	Parietal	Angular Gyrus		**↓**		
R	Parietal	Inferior Parietal Gyrus		**↓**		
R	Parietal	Postcentral Gyrus	**↑**			
R	Temporal	Superior Temporal Gyrus	**↑**			
L	Temporal	Fusiform Gyrus		**↑**		
R	Temporal	Hippocampus		**↓**		
R	Temporal	Middle Occipital Gyrus	**↑**		**↑**	
R	Temporal	Middle Temporal Gyrus		**↓**		
L	Temporal	Parahippocampal Gyrus			**↓**	
R	Temporal	Parahippocampal Gyrus		**↓**		

*Note*: This table summarizes the results of the comparison between ALS patients and healthy controls, stratified by sex, in terms of brain metabolism and brain connectivity. Red cells with ↑ identify brain areas where metabolism or connectivity was increased in patients when compared to controls, while blue cells with ↓ identify brain areas where metabolism or brain connectivity was decreased in patients when compared to controls.

Abbreviations: BIL, bilateral; L, left side; R, right side.

## DISCUSSION

Our study demonstrates that sex has a significant impact on cerebral metabolism and connectivity in ALS. The evidence that such differences cannot be detected when comparing male and female healthy controls suggests that we found a disease‐related effect of sex.

In the direct comparison of brain metabolism between male and female ALS patients, we identified a highly significant relative hypometabolism in males in regions including frontal and occipital cortex. We found similar results in a previous study performed at our centre and comparing ALS patients with HC [28]. This finding could be related to a more extensive degenerative process in males, as supported by the direct comparison of each sex group of patients with respective controls. Another study on a Belgian ALS series similarly reported a frontal hypometabolism compared to HC, but occipital regions resulted to be relatively hypermetabolic [29]. The divergent findings in the occipital regions between the two studies might be related to different patient preparation protocols [30]. Clusters of relative hypermetabolism in ALS patients compared to HC have been consistently reported [28, 29], but the interpretation is challenging, particularly if the cortices giving rise to the corticospinal tracts are involved. A possible explanation is that microglial activation, together with astrocyte reaction, might be a phenomenon underlying the finding of glucose hypermetabolism. This hypothesis is supported by animal models of cerebral ischemia, showing that microglial activation, evidenced by an increased 11C‐PK11195 uptake, is associated with increased ^18^F‐FDG uptake [31]. Moreover, in other neurodegenerative diseases, for example prion diseases, ^18^F‐FDG‐PET hypermetabolism has been interpreted as possibly related to microglial activation [32]. Nevertheless, a conclusive interpretation of brain hypermetabolism in ALS needs further studies based on a double‐tracer approach including radioligands targeting microglia in addition to ^18^F‐FDG. The analyses evaluating the relationship of brain metabolism with motor staging revealed a difference between males and females. Namely, only males showed a positive correlation between corticospinal tracts metabolism and King's stage. As mentioned above, this finding could be due to higher microglial activation, together with astrocyte reaction, in more advanced stages. Otherwise, the analyses evaluating the relationship of brain metabolism with cognitive status showed similar findings in males and females. However, despite the same proportion of Strong categories in the two groups, we found significant differences of the performance in some cognitive tests. Females had worse scores in category fluency, delayed recall of the Rey‐Osterrieth complex figure and in Coloured Progressive Matrices. Otherwise, males had worse performance in the immediate and delayed recall of the Rey Auditory Verbal Learning Test (RAVLT). The association of verbal learning deficits with the relative hypometabolism of parahippocampal gyrus and precuneus in males might be plausible [33]. Nevertheless, the overall interpretation of the observed neuropsychological differences in terms of metabolic changes is likely to be arbitrary, since our study was not specifically designed for this purpose.

The interpretation of the increased connectivity in female as compared to male ALS patients remains challenging. In neurodegenerative diseases, hyperconnectivity of the most functionally central and metabolically efficient regions (i.e. hubs) could be a compensatory mechanism in early phases. On the contrary, long‐term hyperconnectivity may increase metabolic stress of network hubs, making them more vulnerable to degeneration [34]. When increased connectivity is identified in regions with preserved metabolism it may point out the recruitment of compensatory mechanisms. Conversely, when involving metabolically impaired areas, it may indicate the disruption of cerebral coping strategies [35]. Nevertheless, separating these two faces of connectivity changes remains challenging in the absence of longitudinal data [36]. Several hypotheses have been proposed to explain the finding of increased functional connectivity in ALS. Beyond a compensatory phenomenon, it might be related to the pathogenic loss of local inhibitory interneurons [37]. The latter hypothesis is corroborated by PET studies showing a decreased uptake of ^11^C‐flumazenil, a PET tracer targeting benzodiazepine receptor, in the primary motor cortex and frontal cortex [38]. Moreover, studies on mouse models suggest that microglial activation can lead to increased metabolic connectivity [39]. Strikingly, a functional MRI study involving ALS patients, presymptomatic ALS subjects (i.e. carriers of *SOD1* and *C9ORF72* mutations without any neurological symptoms) and healthy controls showed that increased functional connectivity may be among the earliest detectable brain changes in the presymptomatic phase of the disease [40]. In this context, our finding of increased connectivity in female ALS patients in the right cingulate cortex, whose metabolism is relatively spared as compared to males, might be related to a coping strategy towards the degenerative process. In support of this hypothesis, previous studies on ALS have postulated the compensatory role of the cerebellum [41, 42], which resulted to be hyperconnected in our analyses, having a negative significant correlation with the cingulate cortex. The increased connectivity of the right cingulate cortex with the corresponding contralateral region, showing a positive correlation of metabolism, might suggest a possible pathway along which the disease will spread when the compensatory mechanisms will fail, as supported by previous studies about other neurodegenerative diseases [43]. A similar interpretation could underlie our findings of increased connectivity of left superior medial frontal gyrus with occipital (negative correlation of metabolism) and frontal cortices (positive correlation) in females. The comparisons of ALS subjects with HC stratified by sex pointed out the presence of increased connectivity in female ALS as compared to female HC both in metabolically impaired and preserved regions, providing further support to the hypothesis of the coexistence of compensation mechanisms and disruption of coping strategies.

As for the relative hypometabolism in male patients, we can hypothesize that, at a given level of motor and cognitive disability, male patients are able to cope with a higher amount of brain changes related to the disease as compared to females. The factors underlying this resilience can be manifold and can account for its difference in nature between males and females. First, brain reserve (BR) might play a role. According to a threshold model, subjects who initially have a higher BR can tolerate a more consistent depletion of the neurobiological capital before the onset of symptoms [44]. Second, functional reserve (FR) might be involved. Its definition might be derived from that of cognitive reserve, referring to the capability of the brain that allows for a performance that is better than expected given the severity of disease‐related brain damage. A remarkable point for the interpretation of our findings is that FR mechanisms can include molecular and cellular changes, and not only connectivity changes [44]. The idea that male and female ALS patients can have a different FR is supported by studies on healthy ageing and Alzheimer's Dementia [16]. Whether hormonal factors contribute to modulate resilience mechanisms remains unclear. Studies in mice and rats about the effect of gonadectomy and sexual hormone treatments to impact on disease progression showed inconclusive results [8, 9, 10]. However, hormonal factors might modulate neuronal vulnerability by acting over the life course, even in absence of a direct effect on the rate of disease progression. Literature data consistently show a higher incidence of ALS in males compared to females [45]. To provide possible explanations for the effect of sex on ALS incidence, a population‐based study investigated the effect of lifetime endogenous oestrogen exposure showing its impact in reducing ALS risk and increasing survival [46]. Nevertheless, other epidemiological studies [5, 47] showed an increase of ALS incidence in women over time, possibly due to a change in lifestyle, which is progressively becoming similar to that of men (e.g. for cigarette smoking). Therefore, the role of hormonal factors in the modulation of neuronal vulnerability in females should be cautiously considered due to the possible influence of environmental exposures and lifestyle.

Our study has some limitations. First, we do not have longitudinal PET data allowing us to provide a more comprehensive understanding of factors underlying the different mechanisms of brain resilience involved in male and female ALS patients. Second, we cannot completely exclude that some brain metabolic and connectivity differences can be detected in larger samples of healthy individuals, as suggested by other studies [14, 48]. Third, we were not able to compare brain metabolism and networks in females before and after menopause and with or without hormonal replacement therapy. Despite these weaknesses, our work is the first one reporting 2‐[^18^F]FDG‐PET data about sex‐related differences in brain metabolism and connectivity in ALS. Moreover, we can count on a very large ALS sample, fully characterized as regards genetic, motor and cognitive data, thus allowing us to rule out the possible influence of confounders on the results and to compare two groups with the same clinical status. Furthermore, our control group has been recruited in four different centres, suggesting that the lack of sex‐related differences is not centre‐specific.

In conclusion, our study highlights a significant disease‐related effect of sex on cerebral metabolism and connectivity in ALS. We suggest that females and males show different resilience mechanisms to cope with the neurodegenerative process of ALS. Further studies, including longitudinal data, are recommended for a comprehensive understanding of sex‐related variability in ALS, since this issue is of outstanding importance for appropriate design and interpretation of clinical trials in the perspective of precision medicine.

## AUTHOR CONTRIBUTIONS

**Antonio Canosa:** Conceptualization; methodology; software; data curation; investigation; validation; formal analysis; writing – original draft; visualization. **Alessio Martino:** Methodology; software; data curation; investigation; validation; formal analysis; writing – original draft. **Umberto Manera:** Conceptualization; methodology; software; data curation; investigation; validation; formal analysis; visualization; writing – original draft. **Alessandro Giuliani:** Methodology; software; supervision; visualization; writing – review and editing. **Rosario Vasta:** Methodology; data curation; investigation; writing – review and editing. **Francesca Palumbo:** Data curation; investigation; writing – review and editing. **Maurizio Grassano:** Data curation; investigation; writing – review and editing. **Silvia Daniela Morbelli:** Methodology; investigation; visualization; supervision; writing – review and editing. **Matteo Pardini:** Methodology; validation; writing – review and editing; visualization. **Agostino Chiaravalloti:** Methodology; visualization; writing – review and editing; supervision. **Orazio Schillaci:** Methodology; writing – review and editing; visualization; supervision. **Klaus Leonard Leenders:** Writing – review and editing; supervision. **Rosalie Vered Kogan:** Writing – review and editing; data curation; investigation. **Giulia Polverari:** Data curation; investigation; writing – review and editing. **Grazia Zocco:** Data curation; investigation; writing – review and editing. **Francesca Di Pede:** Data curation; investigation; writing – review and editing. **Filippo De Mattei:** Data curation; investigation; writing – review and editing. **Sara Cabras:** Data curation; investigation; writing – review and editing. **Enrico Matteoni:** Data curation; investigation; writing – review and editing. **Cristina Moglia:** Data curation; investigation; supervision; visualization; writing – review and editing. **Andrea Calvo:** Methodology; data curation; investigation; visualization; supervision; resources; project administration; writing – review and editing. **Adriano Chiò:** Visualization; supervision; funding acquisition; project administration; resources; writing – review and editing. **Marco Pagani:** Supervision; funding acquisition; visualization; methodology; data curation; conceptualization; project administration; writing – review and editing.

## FUNDING INFORMATION

This work was supported by the Progetti di Rilevante Interesse Nazionale (PRIN) 2017 (grant 2017SNW5MB) and the Joint Programme‐Neurodegenerative Disease Research (Strength, ALS‐Care and Brain Mend projects), granted by the Italian Ministry of University and Research; a grant from the Thierry Latran Foundation (INSPIRED project); the Italian Ministry of Health (Ricerca Sanitaria Finalizzata, grant RF‐2016‐02362405); the European Commission's Health Seventh Framework Programme (FP7/2007–2013, grant agreement 259867). This study was performed under the Department of Excellence grant of the Italian Ministry of University and Research to the ‘Rita Levi Montalcini’ Department of Neuroscience, University of Turin. The funders had no role in study design, data collection, analysis and interpretation, or writing of the article.

## CONFLICT OF INTEREST STATEMENT

Matteo Pardini reports research support from Novartis and Nutricia and speakers fees from GE, Biogen and Merck. Andrea Calvo has received a research grant from Cytokinetics. Adriano Chiò serves on scientific advisory boards for Mitsubishi Tanabe, Roche, Biogen, Cytokinetics, Denali Therapeutics, Amylyx and AveXis. The other authors report no disclosures relevant to the manuscript.

## STANDARD PROTOCOL APPROVALS, REGISTRATIONS AND PATIENT CONSENTS

All participants signed a written informed consent and the study was approved by the ethical committee of the ‘Azienda Ospedaliero‐Universitaria Città della Salute e della Scienza di Torino’ (Protocol number 0011669).

## Supporting information


Data S1.

## Data Availability

The NIfTI files of the clusters identified in the analyses will be available on demand by interested researchers.
