# Supplementary material for: Sex‐related differences in amyotrophic lateral sclerosis: A 2‐[ 18F]FDG‐PET study
Source: Eur J Neurol. 2024 Dec 10;32(1):e16588. doi: 10.1111/ene.16588 (PMC11629101; doi:10.1111/ene.16588)
Supplement: Supplementary file 1 — Data S1. [file ENE-32-e16588-s001.docx]

**Supplemental Material**

*Supplemental Methods*

We ran separate analyses for male and female patients to evaluate the association between brain metabolism and motor and cognitive staging respectively. In each group King’s stage (1, 2, 3) and cognitive status, classified according to international diagnostic criteria [1], were regressed out against whole-brain metabolism. The SPM12 multiple regression routine was implemented with age at PET, site of onset and cognitive status/King’s stage as covariates. The cognitive categories based on international criteria [1] were graded as follows, based on the results of a previous study [2]: normal cognition = 0, behavioural impairment = 1, cognitive impairment = 2, cognitive and behavioural impairment = 3, frontotemporal dementia = 4. The height threshold was set at p < 0.001 (p < 0.05 FWE corrected at cluster level). In all the analyses, only clusters containing ≥100 contiguous voxels were considered significant. Brodmann areas (BAs) were identified at a 0–2-mm range from the Talairach coordinates of the SPM output isocentres corrected by Talairach Client (http://www.talairach.org/index.html).

**Supplemental Table 1**. Clusters of relative hypometabolism of male patients as compared to male controls. R: right. L: left. BA: Brodmann Area.

| **p(FWE-corrected)** | **Cluster extent** | **Z-score** | **Talairach coordinates (x, y, z)** | | | **Side** | **Brain Region** | **BA** |
| --- | --- | --- | --- | --- | --- | --- | --- | --- |
| 0.000 | 717 | 5.77 | -18 | -21 | -26 | L | Parahippocampal Gyrus | 28 |
|  |  | 5.60 | -16 | -13 | -28 | L | Parahippocampal Gyrus | 35 |
|  |  | 5.49 | -8 | -33 | -5 | L | Cerebellar Culmen | * |
| 0.001 | 104 | 5.40 | 0 | 11 | 62 | L | Superior Frontal Gyrus | 6 |
| 0.001 | 113 | 5.35 | 4 | -58 | 8 | R | Posterior Cingulate | 30 |
| 0.001 | 150 | 5.29 | -32 | 20 | 49 | L | Superior Frontal Gyrus | 8 |
|  |  | 4.57 | -34 | 7 | 53 | L | Middle Frontal Gyrus | 6 |
| 0.002 | 100 | 5.13 | -24 | 34 | -24 | L | Inferior Frontal Gyrus | 11 |
|  |  | 4.96 | -20 | 20 | -20 | L | Inferior Frontal Gyrus | 47 |
| 0.000 | 155 | 4.87 | 0 | -97 | 5 | L | Cuneus | 18 |

**Supplemental Table 2**. Clusters of relative hypermetabolism of male patients as compared to male controls. R: right. L: left. BA: Brodmann Area.

| **p(FWE-corrected)** | **Cluster extent** | **Z-score** | **Talairach coordinates (x, y, z)** | | | **Side** | **Brain Region** | **BA** |
| --- | --- | --- | --- | --- | --- | --- | --- | --- |
| 0.000 | 468 | 5.85 | 57 | -74 | 6 | R | Middle Occipital Gyrus | 19 |
| 0.000 | 232 | 5.66 | 65 | -3 | 19 | R | Precentral Gyrus | 4 |

**Supplemental Table 3**. Clusters of relative hypometabolism of female patients as compared to female controls. R: right. L: left. BA: Brodmann Area.

| **p(FWE-corrected)** | **Cluster extent** | **Z-score** | **Talairach coordinates (x, y, z)** | | | **Side** | **Brain Region** | **BA** |
| --- | --- | --- | --- | --- | --- | --- | --- | --- |
| 0.001 | 158 | 6.05 | -24 | 41 | -24 | L | Superior Frontal Gyrus | 11 |
|  |  | 5.02 | -24 | 28 | -27 | L | Inferior Frontal Gyrus | 11 |
| 0.001 | 160 | 5.74 | -51 | 10 | 40 | L | Middle Frontal Gyrus | 8 |
| 0.000 | 241 | 5.59 | -16 | 0 | -3 | L | Lateral Globus Pallidus | * |
|  |  | 4.83 | -16 | 14 | -23 | L | Inferior Frontal Gyrus | 47 |
|  |  | 4.71 | -12 | 5 | -20 | L | Medial Frontal Gyrus | 25 |
| 0.002 | 102 | 5.57 | 8 | 58 | 32 | R | Superior Frontal Gyrus | 9 |
|  |  | 5.00 | 6 | 68 | 0 | R | Superior Frontal Gyrus | 10 |
|  |  | 4.87 | 6 | 67 | 11 | R | Medial Frontal Gyrus | 10 |

**Supplemental Table 4**. Clusters of relative hypermetabolism of female patients as compared to female controls. R: right. L: left. BA: Brodmann Area.

| **p(FWE-corrected)** | **Cluster extent** | **Z-score** | **Talairach coordinates (x, y, z)** | | | **Side** | **Brain Region** | **BA** |
| --- | --- | --- | --- | --- | --- | --- | --- | --- |
| 0.000 | 326 | 6.05 | 67 | -11 | 23 | R | Postcentral Gyrus | 3 |
| 0.000 | 298 | 5.21 | 53 | -74 | 4 | R | Middle Occipital Gyrus | 19 |
|  |  | 5.21 | 63 | -54 | 16 | R | Superior Temporal Gyrus | 22 |

**Supplemental Table 5**. Results of the node-wise comparison between female ALS cases and female controls.

| **Node** | **Test statistic** | **Nominal p-value** | **q-value** | **Case score** | **Control score** |
| --- | --- | --- | --- | --- | --- |
| Right Angular Gyrus | 15.826 | 0.001 | 0.0085 | 0 | 15.826 |
| Right Middle Cingulum | 2.156 | 0.001 | 0.0085 | 2.156 | 0 |
| Left Inferior frontal Gyrus,  triangular | 7.978 | 0.001 | 0.0085 | 8.726 | 0.748 |
| Left Middle Frontal Gyrus | 9.547 | 0.001 | 0.0085 | 9.547 | 0 |
| Right Middle Frontal Gyrus | 11.914 | 0.001 | 0.0085 | 11.914 | 0 |
| Left Superior Frontal Gyrus | 8.515 | 0.001 | 0.0085 | 9.309 | 0.795 |
| Left Superior Medial  Frontal Gyrus | 15.979 | 0.001 | 0.0085 | 15.979 | 0 |
| Right Superior Medial  Frontal Gyrus | 16.467 | 0.001 | 0.0085 | 17.185 | 0.718 |
| Left Superior Orbital  Frontal Gyrus | 7.982 | 0.001 | 0.0085 | 10.917 | 2.935 |
| Left Lingual Gyrus | 6.382 | 0.001 | 0.0085 | 7.196 | 0.813 |
| Right Parahippocampal  Gyrus | 6.847 | 0.001 | 0.0085 | 1.461 | 8.308 |
| Right Inferior Parietal  Gyrus | 11.13 | 0.003 | 0.0235 | 0.707 | 11.837 |
| Right Calcarine Cortex | 5.454 | 0.004 | 0.0268 | 6.239 | 0.785 |
| Right Hippocampus | 7.146 | 0.004 | 0.0268 | 2.208 | 9.354 |
| Right Inferior Orbital Frontal  Gyrus | 12.355 | 0.005 | 0.0313 | 13.133 | 0.777 |
| Right Anterior Cingulum | 13.006 | 0.006 | 0.0313 | 13.733 | 0.727 |
| Right Lingual Gyrus | 6.126 | 0.006 | 0.0313 | 7.724 | 1.598 |
| Left Middle Occipital Gyrus | 5.629 | 0.006 | 0.0313 | 9.503 | 3.874 |
| Right Precentral Gyrus | 2.344 | 0.008 | 0.0395 | 0 | 2.344 |
| Right Superior Frontal Gyrus | 4.784 | 0.01 | 0.0417 | 6.297 | 1.513 |
| Left Rectus Gyrus | 10.779 | 0.01 | 0.0417 | 13.216 | 2.438 |
| Right Middle Temporal  Gyrus | 12.011 | 0.01 | 0.0417 | 0 | 12.011 |
| Left Calcarine Cortex | 3.132 | 0.011 | 0.0417 | 3.913 | 0.78 |
| Right Middle Orbital Frontal  Gyrus | 10.008 | 0.011 | 0.0417 | 10.776 | 0.768 |
| Left Fusiform Gyrus | 2.929 | 0.012 | 0.0417 | 2.929 | 0 |
| Left Superior Occipital  Gyrus | 7.533 | 0.012 | 0.0417 | 15.448 | 7.915 |
| Right Superior Occipital  Gyrus | 10.54 | 0.012 | 0.0417 | 5.533 | 16.072 |
| Left Inferior Orbital Frontal  Gyrus | 11.777 | 0.013 | 0.0436 | 12.478 | 0.701 |
| Left Inferior Occipital Gyrus | 6.152 | 0.014 | 0.0453 | 7.03 | 0.878 |
| Right Superior Orbital Frontal  Gyrus | 7.437 | 0.015 | 0.047 | 11.166 | 3.729 |

**Supplemental Table 6**. Results of the node-wise comparison between male ALS cases and male controls.

| **Node** | **Test statistic** | **Nominal p-value** | **q-value** | **Case score** | **Control score** |
| --- | --- | --- | --- | --- | --- |
| Left Posterior Cingulum | 0.833 | 0.001 | 0.047 | 0 | 0.833 |
| Right Pallidum | 2.455 | 0.001 | 0.047 | 2.455 | 0 |

**Supplemental Figure 1** Male subjects: clusters whose metabolism decreases as cognitive status worsens are represented in red on axial sections of a brain magnetic resonance imaging template and on the brain surface of a glass brain rendering (bottom right)


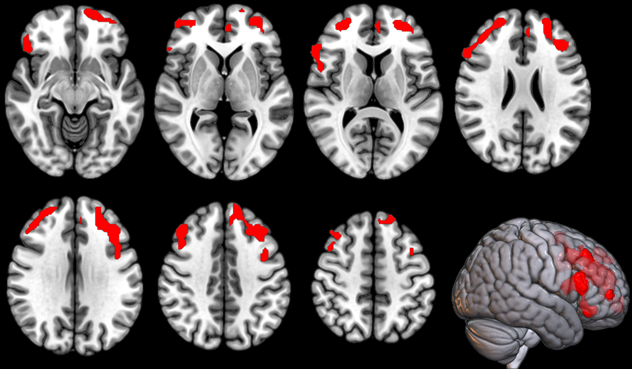


**Supplemental Figure 2** Male subjects: clusters whose metabolism increases as cognitive status worsens are represented on the sections of a brain MRI template


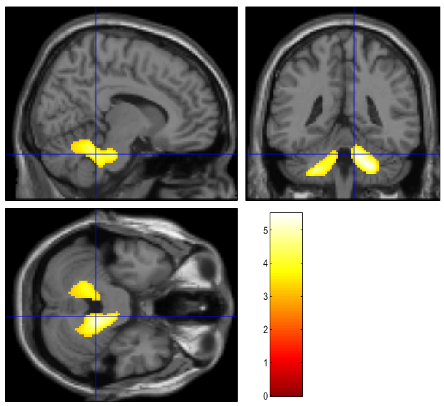


**Supplemental Figure 3** Female subjects: clusters whose metabolism decreases as cognitive status worsens are represented in green on axial sections of a brain magnetic resonance imaging template and on the brain surface of a glass brain rendering (bottom right)

*
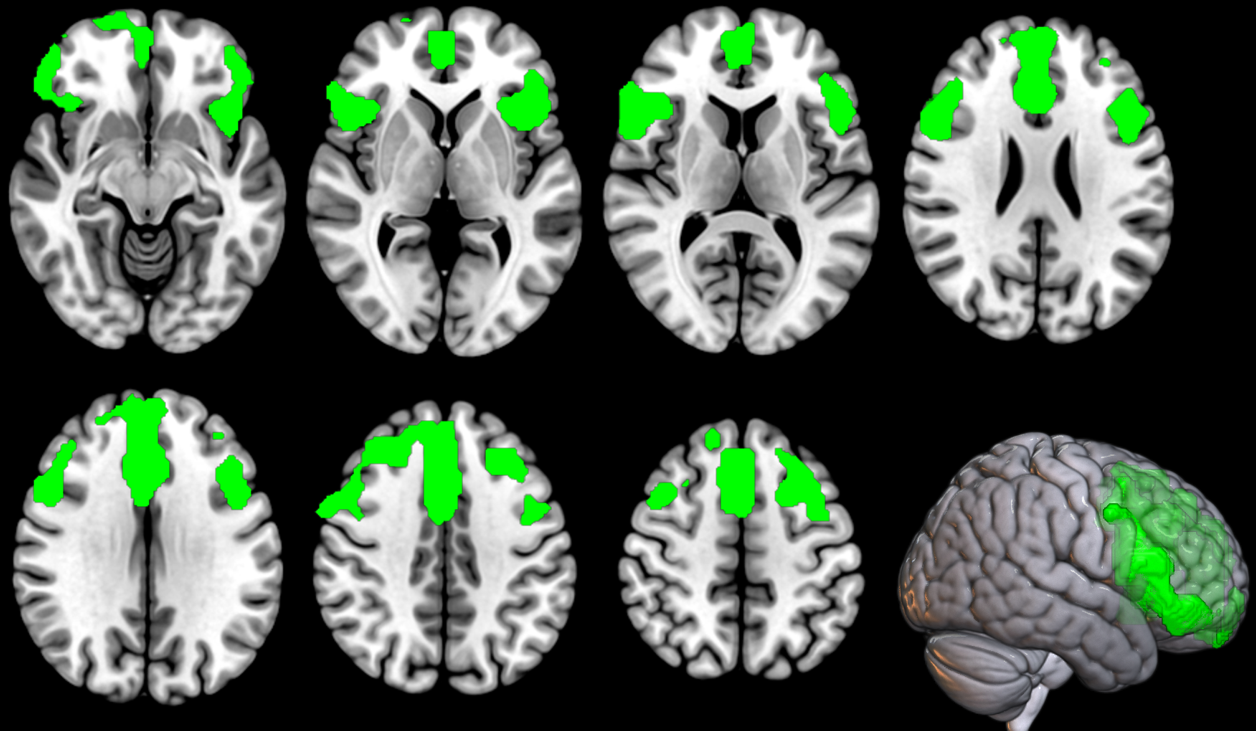
*

**Supplemental Figure 4** Female subjects: clusters whose metabolism increases as cognitive status worsens are represented on the sections of a brain MRI template


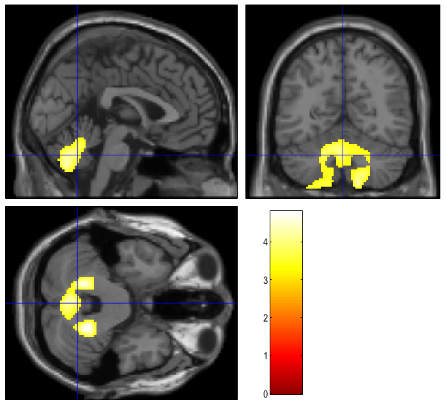


**Supplementary Figure 5** Male subjects: clusters whose metabolism increases as King’s stage worsens are represented on the sections of a brain MRI template

*
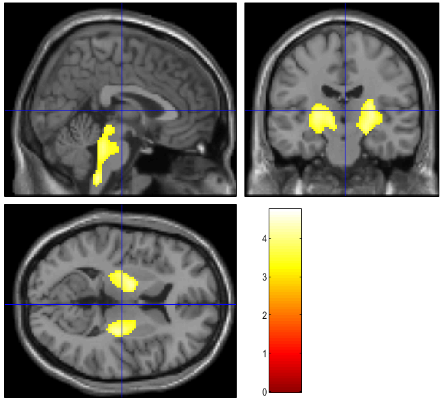
*

**Supplemental Figure 6** Whole-brain metabolic connectivity of female controls is represented on a 3D brain template. The spheres represent the nodes: the higher the size, the higher the metabolic connectivity. Red connections stand for positive correlations, the blue ones for negative correlations


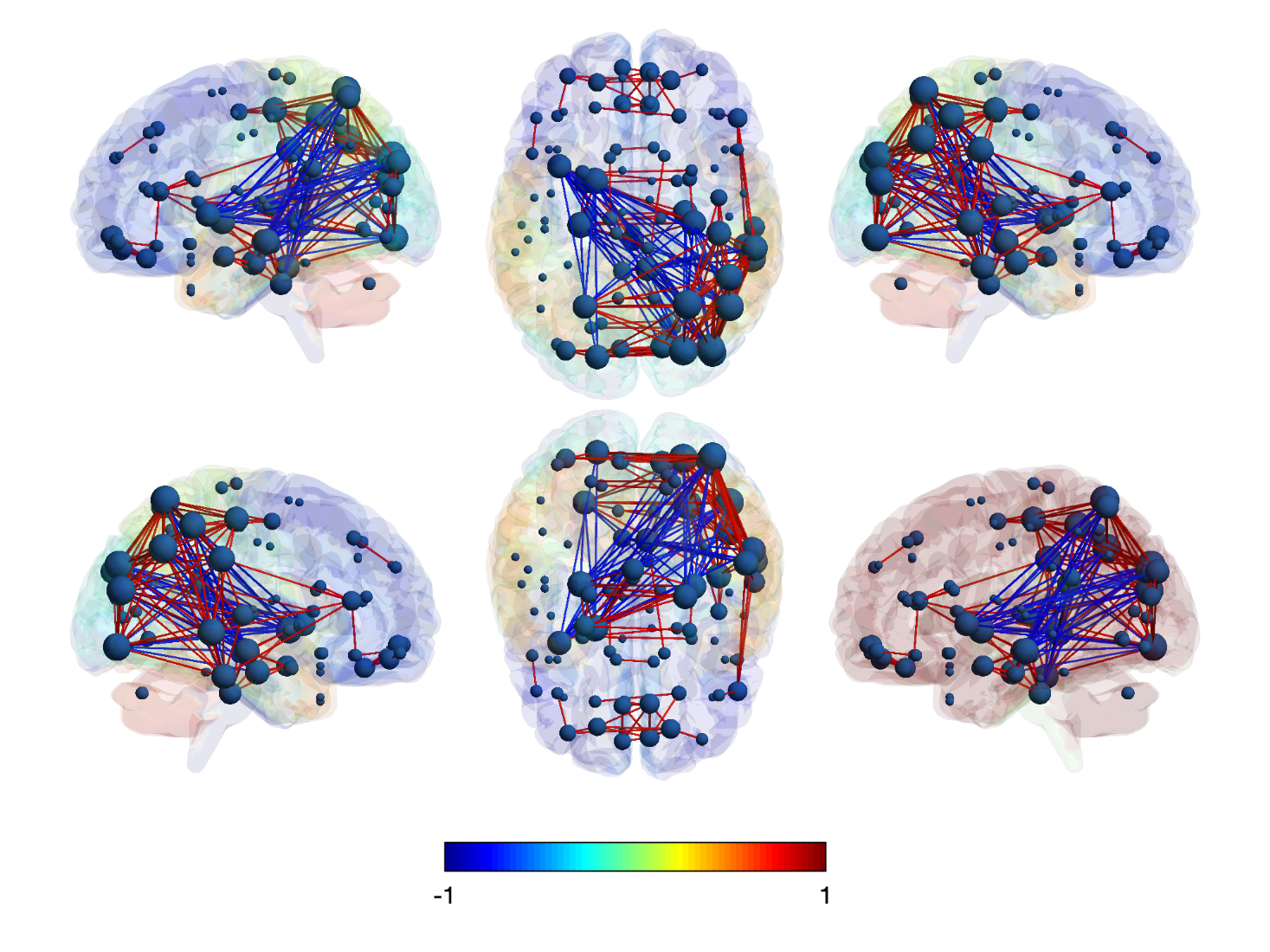


**Supplemental Figure 7** Whole-brain metabolic connectivity of male controls is represented on a 3D brain template. The spheres represent the nodes: the higher the size, the higher the metabolic connectivity. Red connections stand for positive correlations, the blue ones for negative correlations

**
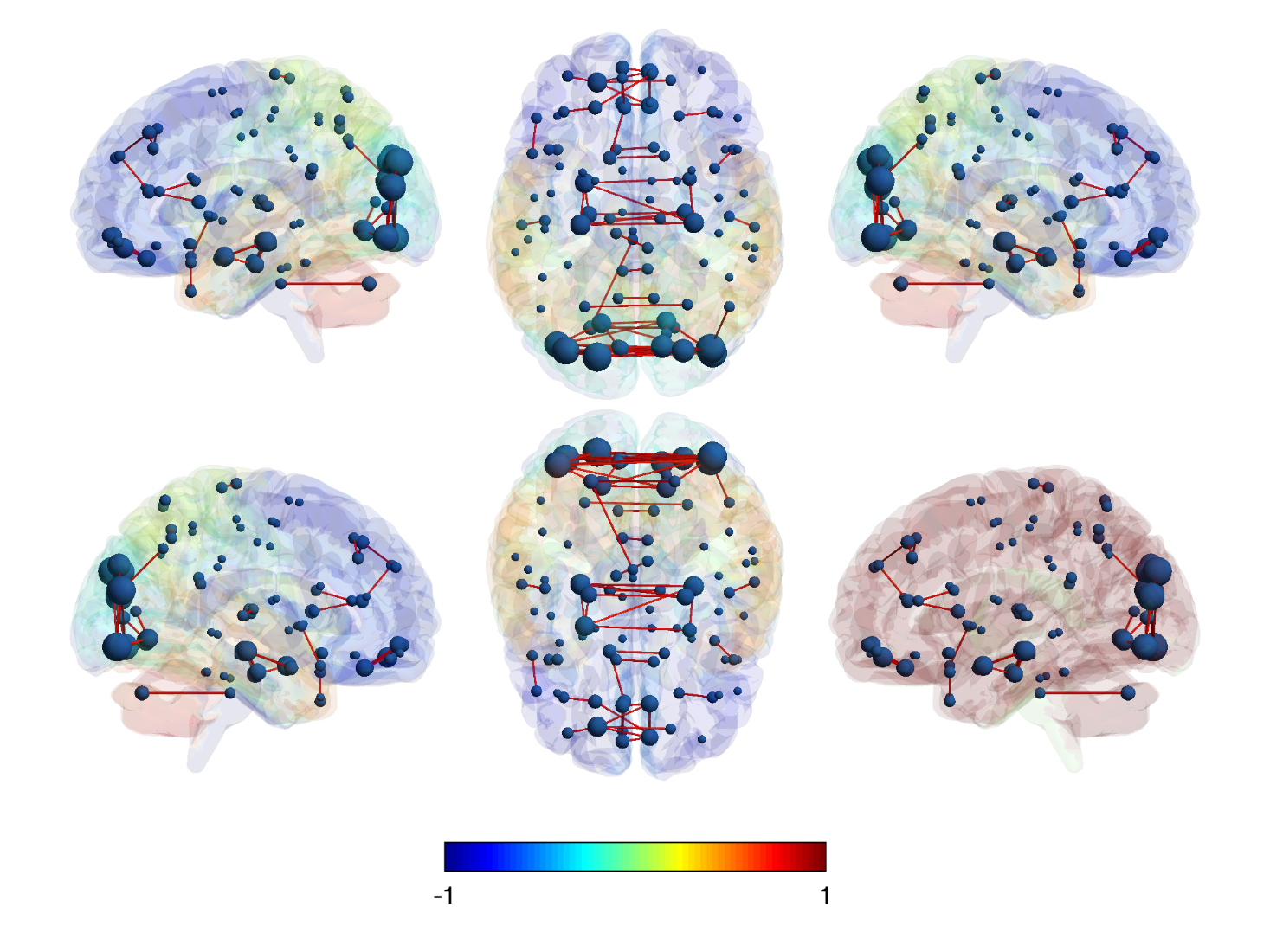
**

**References of supplementary material**

1. Strong MJ, Abrahams S, Goldstein LH, Woolley S, Mclaughlin P, Snowden J, et al. Amyotrophic lateral sclerosis - frontotemporal spectrum disorder (ALS-FTSD): Revised diagnostic criteria. Amyotroph Lateral Scler Front Degener. 2017;18:153–74.
2. Canosa A, Moglia C, Manera U, Vasta R, Torrieri MC, Arena V, et al. Metabolic brain changes across different levels of cognitive impairment in ALS: a ^18^F-FDG-PET study. J Neurol Neurosurg Psychiatry. 2020;jnnp-2020-323876.
